# Favre-Racouchot syndrome associated with eyelid papilloma: a case report

**DOI:** 10.7555/JBR.26.20110093

**Published:** 2012-04-04

**Authors:** Ruzhi Zhang, Wenyuan Zhu

**Affiliations:** a Department of Dermatology, the First Affiliated Hospital of Bengbu Medical College, Bengbu, Anhui 233004, China;; b Department of Dermatology, the First Affiliated Hospital of Nanjing Medical University, Nanjing, Jiangsu 210029, China.

**Keywords:** Favre-Racouchot syndrome, eyelid papilloma, bilateral, comedones, linear

## Abstract

A 55-year-old Chinese man presented with an asymptomatic pedunculated elevation on his left lower eyelid which had been gradually increasing in size during the past three years. The patient was diagnosed with eyelid papilloma by pathological examination. Concomitantly, the patient developed open comedones with a bilateral linear distribution, along with oblique wrinkle lines in his infraorbital regions. These lesions were noninflammatory and remained unchanged for two years. To the best of our knowledge, this distribution of open comedones, especially in combination with eyelid papilloma, has not been reported previously in Favre-Racouchot syndrome.

## INTRODUCTION

Favre-Racouchot syndrome is a disorder consisting of multiple open and closed comedones in the presence of actinically damaged skin[1]. The disease is not an uncommon condition, and it occurs in up to 6% of people older than 50 years of age. The incidence of Favre-Racouchot syndrome increases with age, although it has been reported in patients as early as their second decade of life. Although the exact mechanism of the condition is not known, Favre-Racouchot syndrome has been specifically connected to sun exposure[2], smoking[3] and, in a minority of cases, radiation exposure[4]. The clinical manifestation is characterized by solar elastosis with the presence of nodules, cysts, and comedones. Solar elastosis refers to the damage to dermal elastic tissue due to prolonged exposure to UVA and UVB rays[2]. Interestingly, the comedones found in Favre-Racouchot syndrome are histologically indistinguishable from the primary comedones of acne vulgaris, with the exceptions of a lack of inflammation and the presence of a marked actinic elastosis in the surrounding dermis[5]. Clinically, examination reveals actinically damaged skin with atrophy, yellowish discoloration, wrinkles and furrows, cystic nodules, and punctate, waxy, non-inflamed, soft, open or closed comedones. Favre-Racouchot syndrome can be associated with actinic keratosis, basal and squamous cell carcinoma, cutis rhomboidalis nuchae, trichostasis spinulosa, and keratoacanthoma[6].

Here, we report a case of Favre-Racouchot syndrome with a bilateral linear distribution of open comedones and deep furrows on the face, accompanied by the presence of an eyelid papilloma.

## CASE REPORT

A 55-year-old male was referred to our hospital for an evaluation of an asymptomatic papillary growth on his left lower eyelid, which started three years ago and gradually enlarged over time. Two years ago, a few grouped open comedone-like lesions occurred in both infraorbital regions. These lesions were distributed in a linear pattern and developed along oblique wrinkle lines. They were noninflammatory and remained unchanged for 2 years.

**Fig. 1:**
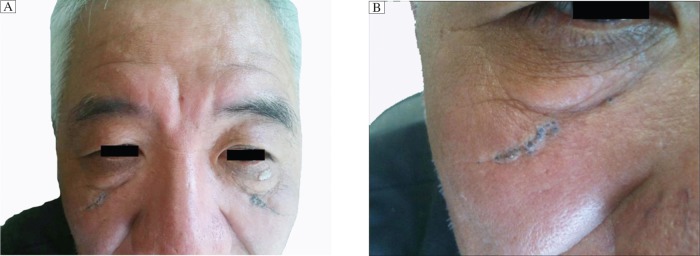
A 55-year old male patient with Favre-Racouchot syndrome. A: Several open comedones are located at two obliquely linear furrows in the infraorbital regions, and an eyelid papilloma is present on the left lower eyelid. B: The open comedones are arranged in deep furrows, and the tip of each horny plug is blackish-grey.

The patient was a farmer who had worked outdoor for many years, which resulted in significant sun exposure. He also had a history of smoking > 10 cigarettes daily for at least 20 years. The patient denied exposure to halogenated aromatic hydrocarbon compounds by inhalation, ingestion, or direct contact of contaminated compounds or foods. No drugs or topical skin care products had been applied on his face.

On physical examination, several open comedones were located at the site of two oblique linear furrows in both infraorbital regions (***Fig. 1A***). The distribution of these lesions was bilaterally symmetrical, although the comedones on the left side appeared grouped. The tip of each horny plug was blackish-grey (***Fig. 1B***). The plug could be expressed with some difficulty from the follicular orifice. No inflammation was present, unlike the comedones seen in acne vulgaris. Simultaneously, a flesh-colored nipple-like growth, approximately the size of groundnut kernels, was observed on the left lower eyelid (***Fig. 1A***). Marked actinically damaged skin with yellowish discoloration, yellowish nodules, atrophy, wrinkles, and furrows was present. No other comedones were detected and the remainder of his physical examination was unremarkable.

**Fig. 2:**
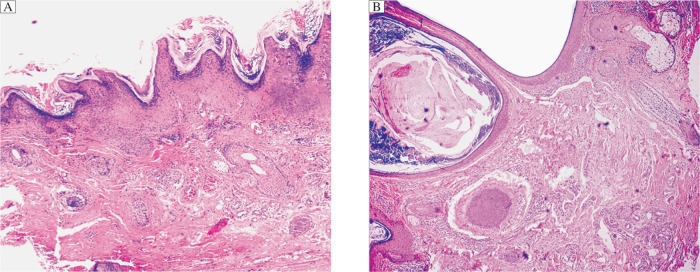
Histological examination of the skin of a patient with Favre-Racouchot syndrome(H&E staining, ×100). A: hypergranulosis and acnthosis with multiple follicular keratinous plugs. B: The infundibula are dilated and elongated and are filled with keratosic and parakeratotic materials. The upper part of the comedone contains yeast-like organisms (pityrosporum) and bacteria. The sections demonstrate that oblique cuts through open comedones may create the false impression of cysts.

An excisional biopsy of the nipple-like growth revealed hyperkeratesis, hypergranulosis and acanthosis with multiple follicular keratinous plugs. The rete ridges were elongated and the upward proliferation of papillae looked like papilomatosis (***Fig. 2A***). One comedone from the right infraorbital area was biopsied, fixed in formalin, serially sectioned and stained with hematoxylin and eosin. The pathological examination showed that dilated and elongated infundibula were filled with keratosic and parakeratotic materials. The comedone wall was intact, though thin. The upper part of the comedo contained yeast-like organisms (pityrosporum) and bacteria. The sections illustrated that oblique cuts through open comedones may create the false impression of cysts (***Fig. 2B***). Based on clinical and histopathological findings, we diagnosed the patient with Favre-Racouchot syndrome associated with eyelid papilloma. The eyelid papilloma was excised for pathological examination. The contents of the comedones were squeezed out gently using a commercially available “comedo expressor.” The patient was told to avoid sun exposure, particularly between 10:00 am and 2:00 pm, and to stop smoking.

Written informed consent was obtained from the patient for publication of this case report and accompanying images.

## DISCUSSION

As advances in medical care have prolonged lifespan and expanded the elderly population, there is a need to evaluate various cutaneous disorders in the growing geriatric population. Solar or senile comedones are a relatively common finding among elderly patients who have had prolonged exposure to solar radiation. The clinical manifestations of multiple open and closed comedones, with yellowish nodules of elastotic materials in a middle-aged to elderly indivi-dual are sufficient for the diagnosis of Favre-Racouchot syndrome. Favre-Racouchot most commonly presents in elderly white men with a history of long-term sun exposure, heavy smoking and, although rare, a history of radiation exposure[2]–[4].

It is important that all patients be advised to take proper sun precautions, such as wearing sunscreen with a sun protective factor of at least 30 with UVA and UVB protection and avoiding outdoors activities between 10:00 am and 2:00 pm if at all possible. If the patient is a smoker, smoking cessation is strongly advised[3]. In treating the patient, different approaches, e.g., medication and surgery, can be employed[7]. Topical retinoids, such as tretinoin, adapalene, or tazarotene, are the most effective pharmacologic treatments. Surgical techniques include excision, dermabrasion, curettage[8], comedone extraction, and laser resurfacing. Although these techniques have yielded poor results when used independently, they provide patients with Favre-Racouchot syndrome a very favorable outcome when used in conjunction with medication.

This case is of great interest for two reasons: First, the open comedones were present in two oblique lines, along with deep furrows[8], which are usually straight lines that begin to appear on the faces of aging people. To the best of our knowledge, most cases of Favre-Racouchot syndrome present with multiple open and closed comedones in the periorbital and temporal areas[9]. The uncommon clinical characteristic in this case has not been previously reported in China. Concurrently, hallmark signs of facial aging, including lines, wrinkles[9], folds and furrows, were observed on his face. The facial features met the diagnostic criteria of Favre-Racouchot syndrome. Markedly reduced collagen content due to chronic sun exposure has been believed to be responsible for severe wrinkle formation in photo-aged skin of the elderly. It is suggested that the elastotic fibers may contribute to a change in the supporting function of the dermis and secondary sebum retention and comedo formation.

Secondly, the patient developed an eyelid papilloma. Goldberg and Altman[6] highlighted the importance of sun exposure in the pathogenesis of periorbital comedones and underscored the frequent association with actinic keratosis, cutis rhomboidalis and epitheliomas. Its association with eyelid papilloma has not been reported so far. Eyelid papilloma is the most common benign eyelid lesion[10]. The exact etiology is unclear. They represent a benign hyperplasia of the surface epithelium and may be sessile or pedunculated. They mainly occur in middle-aged and elderly individuals and may be solitary or multiple, occurring anywhere on the eyelid. They differ from infective warts, which consist of inflammatory hypertrophy of surface epithelium with viral inclusions. Treatment of the condition includes surgical excision or laser ablation. Incidence of the disease increases steadily with age, but it may occur at any age and is seen most frequently in patients older than 30 years. Physical examination of the skin for additional lesions and palpation of the preauricular and submaxillary lymph nodes for metastasis should be conducted if a malignant lesion is suspected[11].

In summary, the presence of papilloma and open comedones can be explained by solar elastosis due to chronic sun damage. Notably, the open comedones are grouped and arranged in two symmetrically oblique lines along with facial deep furrows, which are considered to be the hallmarks of facial aging.
